# Spouse-to-Spouse Transmission and Evolution of Hypervariable Region 1 and 5’ Untranslated Region of Hepatitis C Virus Analyzed by Next-Generation Sequencing

**DOI:** 10.1371/journal.pone.0150311

**Published:** 2016-02-26

**Authors:** Kamila Caraballo Cortes, Osvaldo Zagordi, Joanna Jabłońska, Agnieszka Pawełczyk, Natalia Kubisa, Karol Perlejewski, Iwona Bukowska-Ośko, Rafał Płoski, Marek Radkowski, Tomasz Laskus

**Affiliations:** 1 Department of Immunopathology of Infectious and Parasitic Diseases, Medical University of Warsaw, Warsaw, Poland; 2 Institute of Medical Virology, University of Zurich, Zurich, Switzerland; 3 Clinics of Infectious, Tropical Diseases and Hepatology, Medical University of Warsaw, Warsaw, Poland; 4 Department of Medical Genetics, Medical University of Warsaw, Warsaw, Poland; University of Alberta, CANADA

## Abstract

Hepatitis C virus (HCV) transmission between spouses remains poorly characterized, largely due to the limited availability of samples from the early stage of infection, as well as methodological constraints. A fifty-eight year-old male developed acute hepatitis C infection and his 53-year old spouse has been HCV-positive for over 10 years. Serum samples were collected from both at the time of acute hepatitis C diagnosis in male (baseline) and then at 9 and 13 months. Hypervariable region 1 (HVR1) and 5’ untranslated region (5’UTR) sequences were amplified and subjected to next generation sequencing (NGS) using a pyrosequencing platform. Genetic variants were inferred by Shorah reconstruction method and compared by phylogenetic and sequence diversity analysis. As the sequencing error of the procedure was previously determined to be ≤ 1.5%, the analysis was conducted with and without the 1.5% cut-off with regard to the frequency of variants. No identical HVR1 variants were identified in spouses at baseline and follow-up samples regardless whether the cut-off was applied or not. However, there was high similarity (98.3%) between a minor baseline donor variant (1.7% frequency) and the most abundant baseline recipient variant (62.5% frequency). Furthermore, donor and recipient strains clustered together when compared to 10 control subjects from the same area and infected with the same HCV subtype. There was an increase in HVR1 complexity (number of genetic variants) over time in both spouses. In contrast, the 5'UTR region was stable and of low complexity throughout the study. In conclusion, intrafamilial HCV transmission may be established by a very minor variant and investigation of this phenomenon requires high-sensitivity assays, such as NGS.

## Introduction

Characterization of hepatitis C virus (HCV) transmission patterns remains challenging due to the long incubation periods, asymptomatic course of infection and the scarcity of samples from donor and recipient around the time of transmission [1, 2]. In up to 40% of HCV infections, no clear risk factor can be identified [3].

Horizontal transmission of HCV from spouse to spouse has been shown in a number of publications [4–8]. Such studies require HCV sequence comparison followed by phylogenetic analysis to verify the common ancestry of HCV strains [4]. However, HCV similarity assessment has usually been done by analyzing the consensus sequence only, and such an approach does not take into account high viral diversity [9]. Analysis of the entire spectrum of genetic variants (quasispecies) could be more informative particularly in the light of the high turnover rate of the virus, which may result in rapid changes in the spectrum of circulating viral variants. Furthermore, infection of the recipient is often initiated by a single donor variant [10].

Next-generation sequencing (NGS) techniques, which allow for the evaluation of a wide spectrum of quasispecies including minor variants, is uniquely suited for the investigation of transmission [11]. This approach has been successfully applied in a wide range of viral analyses including human papilloma virus (HPV) genotyping, characterization of HCV and human immunodeficiency virus (HIV) quasispecies, and detection of minor drug-resistant HIV, HCV, and hepatitis B virus (HBV) variants [12–15].

Recently, several studies approached in-depth analysis of hepatitis C virus transmission patterns, using single-genome or next-generation sequencing [16, 17]. However, these studies were focused on molecular identification of putative transmitter/founder variants and not on comparing viral populations of donor and recipient [18, 19].

The aim of the current study was to use NGS to analyze transmission and selection of HCV variants from chronically infected female spouse (donor) to her male spouse (recipient) who subsequently developed acute infection and chronic hepatitis C. We studied two different HCV genomic regions: HVR1 (hypervariable region 1) and 5’UTR (5’ untranslated region). HVR1 is a highly exposed fragment of envelope 2 glycoprotein and a major target for specific antiviral response and its variability facilitates immune evasion and reflects the immune pressure of the host [20]. In contrast, the highly conserved 5’UTR is a non-coding region harboring internal ribosomal entry site (IRES) and its variability impacts the efficiency of translation [21, 22]. Our study provides evidence for the selective transmission of a minor HCV variant and its subsequent rapid molecular evolution in the recipient.

## Materials and Methods

The study involved serum samples from a 53-year old female (donor) who had documented chronic hepatitis C for 10 years (most likely due to iatrogenic infection) and her 58-year old male spouse who developed an acute infection evolving to chronic hepatitis (recipient). Both were infected by the same subtype 1b and samples were collected at baseline (October 2012), which was the time of acute infection in the male (month 0) and after 9 and 13 months. Some clinical and virological data on both spouses are presented in Table 1.

**Table 1:** Clinical and Virological Characteristics of Female and Male Spouses Infected with Hepatitis C Virus.

	Female (chronic hepatitis C)			Male (acute hepatitis C evolving to chronic hepatitis C)^c^		
Time	baseline	9 months	13 months	baseline	9 months	13 months
Age (years)	53			58		
Alanine aminotransferase levels [U L^-1^]; ref. values: 10–40 U L^-1^	50	58	69	1447	>2000	>1000
Liver histology^a^ staging			F0/F1			F1
Viral load [IU mL^-1^]^b^	0.23×10^6^	1.43×10^6^	5.24×10^6^	0.21×10^6^	0.27×10^6^	4.13×10^6^

^a^METAVIR Histologic Scoring System [23].

^b^Roche Cobas Taqman HCV assay (Roche, Indianapolis, IN, USA).

^c^Patient was treated for acute HCV infection with interferon alfa-2b (Intron A, Schering Plough Corporation, Kenilworth, NJ, USA) for 6 months right after baseline sample collection.

Following recommendations the male spouse was treated for 6 months with interferon alfa-2b (Intron A, Schering Plough Corporation, Kenilworth, NJ, USA) 3 mln IU given daily for the first 4 weeks and three times per week thereafter for 20 weeks, but without success (Table 1). Except for the exposure to infected spouse, no other risk factors were identified.

Sera from 10 randomly selected, chronic hepatitis C patients were used as controls for phylogenetic and genetic distance analysis. These patients were of similar age, were infected with same HCV subtype, and were recruited at the same time as the study subjects. The study was approved by the Bioethical Committee of the Medical University of Warsaw (Approval Number KB/17/2013) and all patients provided written informed consent.

### HVR1 and 5’UTR amplification

HVR1 and 5’UTR amplifications were performed as described previously [24, 25]. In brief, total RNA was extracted from 250 μl of serum by a modified guanidinium thiocyanate-phenol/chlorophorm method using Trizol (Life Technologies, Carlsbad, CA, USA). RNA was subjected to reverse transcription at 37°C for 30 minutes using AccuScript High Fidelity Reverse Transcriptase (Agilent Technologies, Santa Clara, CA, USA). A region of 175 nt encompassing HVR1 and 250 nt covering 5’UTR were amplified in two-step PCR using FastStart High Fidelity Taq DNA Polymerase (Roche, Indianapolis, IN, USA). Primers used for reverse transcription and first round HCV HVR1 amplification were as follows: 5′-CATTGCAGTTCAGGGCCGTGCTA-3′ (nt 1632–1610) and 5′-GGTGCTCACTGGGGAGTCCT-3′ (nt 1389–1408). Primers used for reverse transcription and first round HCV 5’UTR amplification were as follows: 5’-TGRTGCACGGTCTACGAGACCTC-3’ (nt 342–320) and 5’-RAYCACTCCCCTGTGAGGAAC-3’ (nt 33–55). Primers employed in the second round PCR contained tags recognized by GS Junior sequencing platform, standard 10-nucleotide multiplex identifiers and target-complementary sequence [24]. Target-complementary sequences of primers for the second round PCR for HVR1 amplification were as follows: 5′- TCCATGGTGGGGAACTGGGC-3′ (positions 1428–1447) and 5′-TGCCAACTGCCA TTGGTGTT-3′ (nt 1603–1584). Target-complementary sequences of primers for the second round PCR for 5’UTR amplification were as follows: 5’-ACTGTCTTCACGCAGAAAGCGTC-3’ (nt 57–79) and 5’-CAAGCACCCTATCAGGCAGTACC-3’ (nt 307–285).

### Pyrosequencing

The amount of DNA equivalent to 3×10^7^ amplicons was subjected to emulsion PCR using GS Junior Titanium emPCR Lib-A Kit (454 Life Sciences, Branford, CT, USA). Pyrosequencing was carried out according to the manufacturer’s protocol for amplicons using GS Junior System (454 Life Sciences).

### Data analysis

Sequencing errors (mismatches, insertions and deletions) were corrected and haplotypes inferred using the program diri_sampler from the Shorah software (https://www1.ethz.ch/bsse/cbg/software/shorah) [26]. Haplotypes that had posterior probability > 95% and were represented by at least 10 reads were extracted with LStructure (https://github.com/ozagordi/LocalVariants/blob/master/src/LStructure.py).

Additionally, although error correction allows for a reliable estimation of variants at a lower frequency, we applied a 1.5% frequency cut-off to improve the specificity of our analysis [27]. As demonstrated in our earlier study based on sequencing of cloned HVR1 sequences, this particular cut-off value corresponds to the aggregate error of amplification and sequencing with the GS Junior platform [27]. Subsequently, 5’UTR and HVR1 haplotypes were aligned to the 1b HCV reference sequences (GenBank accession number AJ242654 and AJ406073, respectively) and the latter was translated into amino acid sequences by MEGA (*Molecular Evolutionary Genetics Analysis)*, version 5.0 (http://www.megasoftware.net/) [28]. Phylogenetic trees of both regions were constructed according to the Maximum Likelihood method based on the Tamura-Nei model [29] using MEGA 5.0. Molecular clock analysis was performed in MEGA 5.0. Genetic diversity and distance parameters were assessed by DNA SP version 5 (http://www.ub.edu/dnasp/) and MEGA 5.0. Sequence similarity was compared using Clustal 2.1 Percent Identity Matrix (http://www.clustal.org/omega/) [30]. Amino acid sequence logos were generated by Web Logo (http://weblogo.berkeley.edu/) [31].

## Results

Altogether, 71,923 reads were obtained from 6 analyzed samples, 13,410 for HVR1 and 58,513 for 5’UTR region. After reconstruction, 7 to 49 variants were inferred per sample for HVR1 and from 6 to 10 for 5’UTR. Application of the cut-off lowered the number of inferred variants as there were now 4 to 20 variants per sample for the HVR1 and from 1 to 2 for the 5’UTR. For the unrelated 10 chronic hepatitis C patients, 33,810 HVR1 reads were obtained (4 to 16 variants per sample).

### HVR1 sequence variability

#### Similarity between spouses

No identical HVR1 variants were identified to be present in both spouses (either above or below the 1.5% cut-off value), but a minor baseline donor variant (1.7% frequency) was found to be closely related to all recipient variants present at baseline (97.1%–98.3% sequence similarity). Furthermore, recipient consensus sequence at baseline differed only by two nucleotide substitutions and one insertion when compared to the putative infecting variant of 1.7% frequency. Only one amino acid difference was present between the infecting minor variant and the recipient major variant (substitution S to A within the epitope for neutralizing antibodies, described below as Epitope 1 [32]).

#### Recipient male spouse

When employing the 1.5% cut-off, the number of variants in the male recipient increased from four (baseline) to five at month 9 and to seven at month 13. At baseline, the HVR1 population was composed of one predominant variant (62.5%), one variant of 24.7% frequency and two minor (defined as <10% frequency) variants. Sequence similarity of the predominant variant to other baseline variants was from 97.7%, to 98.9%. None of the variants present in the initial sample were found in the two follow-up samples, and only one variant was present both at 9 and 13 months (constituting 76.0% and 2.8% of the total, respectively). At 9 months, the frequency of predominant variant was 76.0% but at month 13 the population became more dispersed, with two most abundant variants constituting 52.8% and 23.0% of the population, and five minor variants. Nucleotide diversities per site within HVR1 populations are shown in Table 2. Genetic distances between HVR1 populations (intrahost) were 0.020 (baseline and month 9), 0.028 (month 9 and month 13) and 0.021 (baseline and month 13).

**Table 2:** Nucleotide Diversity Parameters of 5’UTR and HVR1 HCV Variants in Analyzed Spouses.

	5’UTR						HVR1					
Time	Baseline (month 0)		Month 9		Month 13		Baseline (month 0)		Month 9		Month 13	
Analysis of variants	Cut-off 1.5%	No cut-off	Cut-off 1.5%	No cut-off	Cut-off 1.5%	No cut-off	Cut-off 1.5%	No cut-off	Cut-off 1.5%	No cut-off	Cut-off 1.5%	No cut-off
Number of variants in female (donor)	2	9	2	9	2	8	14	41	19	38	20	49
Number of variants in male (recipient)	1	10	1	6	1	7	4	7	5	11	7	17
Number of nucleotide substitutions^a^ in female (donor)	1	2	1	5	1	1	50	55	56	52	55	50
Number of nucleotide substitutions^a^ in male (recipient)	1	3	1	1	1	3	35	35	37	45	38	39
Nucleotide diversity per site^a^ in female (donor)	0.003	0.002	0.003	0.006	0.003	0.002	0.066	0.048	0.067	0.047	0.061	0.044
Nucleotide diversity per site^a^ in male (recipient)	0.004	0.002	0.004	0.001	0.004	0.004	0.082	0.055	0.080	0.056	0.062	0.038

^a^With respect to AJ242654 (for 5’UTR) and AJ406073 (for HVR1) reference sequences.

**Fig 1:**
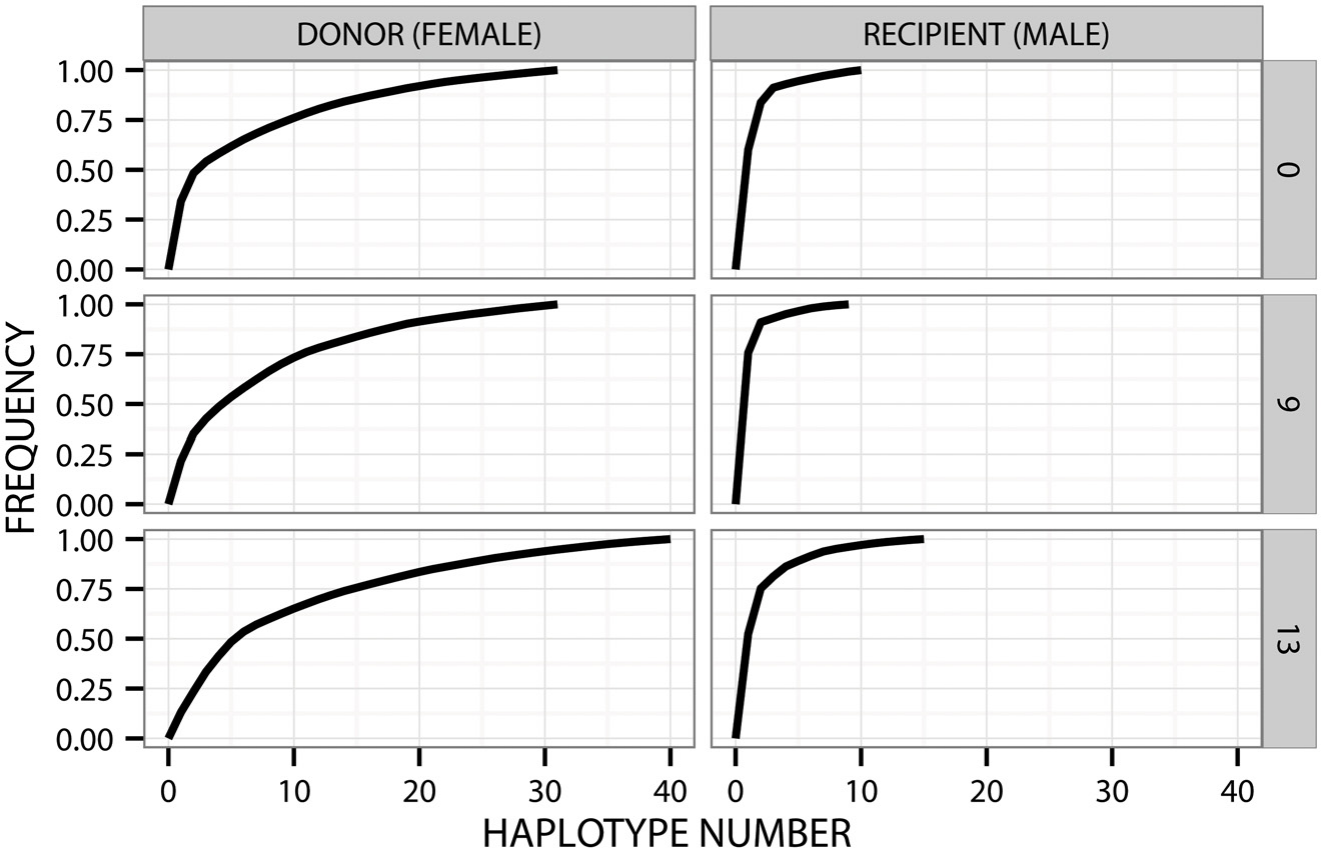
HVR1 quasispecies profile in both spouses at three different time points (0, 9 and 13 months). On the X axis are the haplotypes ordered by decreasing frequency, while cumulative distribution is shown on Y axis. The flatter the curve, the more complex the quasispecies, with more haplotypes within the population. The male spouse was treated for 6 months with interferon starting immediately after the baseline serum was drawn, but the therapy was ultimately unsuccessful. Presented are haplotypes that had posterior probability > 95% and represent at least 10 reads.

Without the cut-off, the number of variants in the recipient increased from seven (baseline) to 11 at month 9 and 17 at month 13. At baseline, HVR1 population was composed of one predominant variant (62.5%), one variant of 24.7% frequency and five minor variants (defined as <10% frequency). Sequence similarity of the predominant variant to other baseline variants ranged from 97.1%, to 99.4%. None of the variants present in the initial sample was found in the follow-up samples, and only one variant was present both at month 9 and 13 (constituting 76.0% and 2.8%, respectively). At 9 months the frequency of predominant variant was 76.0% but at month 13 the population became more dispersed, with the two most abundant variants constituting 52.8% and 23.0% of the population, and 15 minor variants. These populations gave rise to a steep curve on the cumulative distribution plots (Fig 1). Nucleotide diversities per site within HVR1 populations are presented in Table 2. Genetic distances between HVR1 populations (intrahost) were 0.017 (baseline and month 9), 0.029 (month 9 and month 13) and 0.019 (baseline and month 13).

#### Donor female spouse

The number of variants above the cut-off value of 1.5% increased in the donor during the follow-up from 14 (baseline) to 19 (month 9) and 20 (month 13). The baseline HCV population consisted of one predominant variant (33.2%), followed by a variant representing 13.6% and 12 minor variants. At 9 months, the contribution of minor variants increased to 17, while the frequency of the major variant declined to 20.9%. At 13 month, the population was even more dispersed with the most abundant variant constituting only 12.8% of the population and 18 different variants of lower frequency. Seven of the baseline variants were also present at month 9 (36.8% of population) and six baseline variants were still detectable at month 13 sample (30.0% of variants). The frequency of variants found in at least two donor samples ranged from 1.5% to 33.2%. Nucleotide diversity per site within HVR1 in the donor are presented in Table 2. Intrahost genetic distances between HVR1 populations were 0.051 (baseline *vs* month 9), 0.048 (month 9 *vs* month 13) and 0.050 (baseline *vs* month 13).

Genetic distance between donor’s and recipient’s baseline populations (interhost) was 0.054, remained the same at month 9 and increased to 0.057 at month 13.

When conducting the analysis without the cut-off, the number of variants in the donor fluctuated during the follow-up from 41 (baseline) to 38 (month 9) and 49 (month 13). The baseline HCV population consisted of one predominant variant (33.2%), followed by a variant representing 13.6% and 39 minor variants (< 10% each). At 9 months, the number of minor variants was 37, while the proportion of the major variant decreased to 20.9%. At 13 months the population was even more dispersed with the most abundant variant constituting only 12.8% of the population and the presence of 48 different minor variants. These populations resulted in a flatter curves on the cumulative distribution plots when compared to the recipient spouse (Fig 1). Eighteen of the baseline variants were also present at month 9 (47.4% of population) and 13 were still detectable at month 13 (26.5% of all variants). Variants found in at least two donor samples had frequencies of 0.3% to 33.2%. Nucleotide diversities per site in the donor are presented in Table 2. Intrahost genetic distances between HVR1 populations were 0.038 (baseline *vs* month 9), 0.041 (month 9 *vs* month 13) and 0.040 (baseline *vs* month 13).

Genetic distance between donor’s and recipient’s populations (interhost) were 0.037 at baseline, 0.041 at 9 months, and increased to 0.053 at 13 months.

### 5’UTR sequence variability

#### Recipient male spouse

When conducting the analysis with 1.5% cut-off, one predominant HCV variant, which was identical to one of donor baseline variants, was present in all samples (92.3%, 95.7% and 95.9% frequency).

Conducting the analysis without the cut-off did not change the frequency of the predominant variant, but there were now nine minor variants at baseline, five at 9 months and six at 13 months. Their frequency ranged from 0.1% to 1.4% of the total population.

#### Donor female spouse

Two variants of similar frequency (46.5% and 51.0%) were present in the donor spouse serum at baseline and during the follow-up period (49.4% and 47.2% at month 9); (54.4% and 41.7% at month 13). Interhost genetic distance between baseline populations was 0.002, and remained the same at month 9 and 13.

When conducting the analysis without the cut-off, the above two dominant variants were present at identical frequency at baseline (46.5% and 51.0%), at 9 months (49.4% and 47.2%), and at 13 months (54.4% and 41.7%). However, there were now also minority variants present: seven at baseline, seven at 9 months, and six at 13 months, ranging in frequence from 0.1% to 1.1%. Genetic distance between baseline populations (interhost) was 0.007, 0.006 at month 9 and 0.004 at month 13.

Minor variants in both spouses mostly differed by deletions and/or insertions in the homopolymeric cytosine region within 5’UTR (positions 127–129 of the AJ242654 reference genome) and at positions 66–71 (AJ242654 reference genome).

### Phylogenetic analysis of HVR1 variants

**Fig 2:**
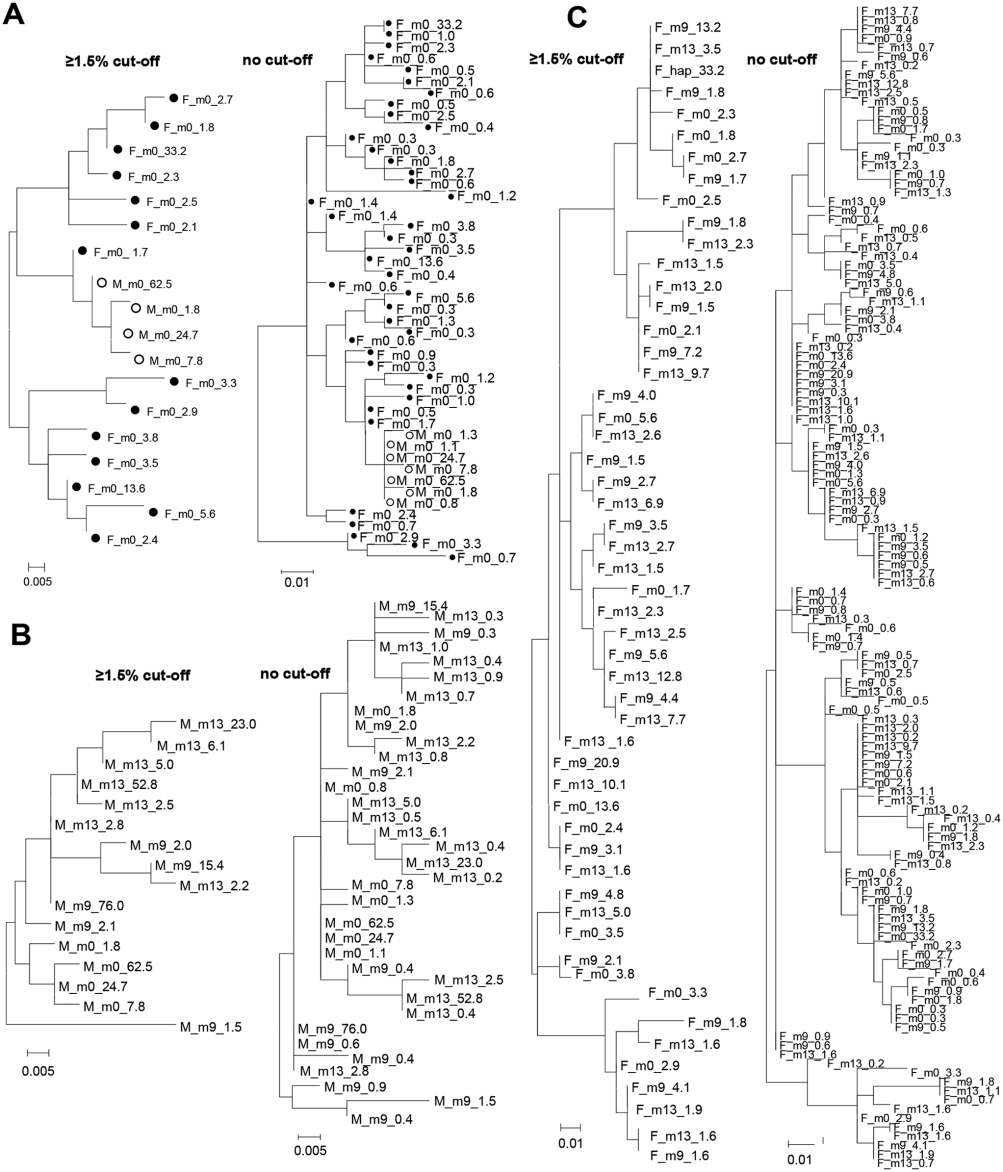
Phylogenetic analysis of HVR1 variants. (A) Variants present in serum at baseline (m0) in the female donor (F, black dot) and recipient male (M, open circle); (B) Variants present in serum over time of observation (m0, month 0; m9, month 9; m13, month 13) in the recipient (M); ultimately unsuccessful interferon monotherapy was given for six months starting immediately after drawing the baseline (m0) sample (C) variants present in the female donor (F) serum at baseline (m0), 9 months (m9) and 13 months (m13). Left panels show variants ≥ 1.5% cut-off, whereas the right panels show all reconstructed variants. Variant frequencies are expressed as percent values and follow time point of sample collection. The evolutionary history was inferred by using the Maximum Likelihood method based on the Tamura-Nei model [29]. Evolutionary analyses were conducted using MEGA 5.0 [28].

Phylogenetic analysis revealed that HVR1 recipient variants were highly similar to one minor donor variant of 1.7% frequency (Fig 2). Based on the estimated mutation rate of HVR1 sequence (8.6 × 10^−2^ substitutions per site per year, [33]), molecular clock analysis suggested that the major recipient (62.5%) variant had a common ancestor with the putative infecting variant of 1.7% frequency approximately 1.5 months prior to baseline sample. Furthermore, there was no clustering of variants from any particular time point.

#### Phylogenetic comparison of HVR1 with non-related chronic hepatitis C patients

**Fig 3:**
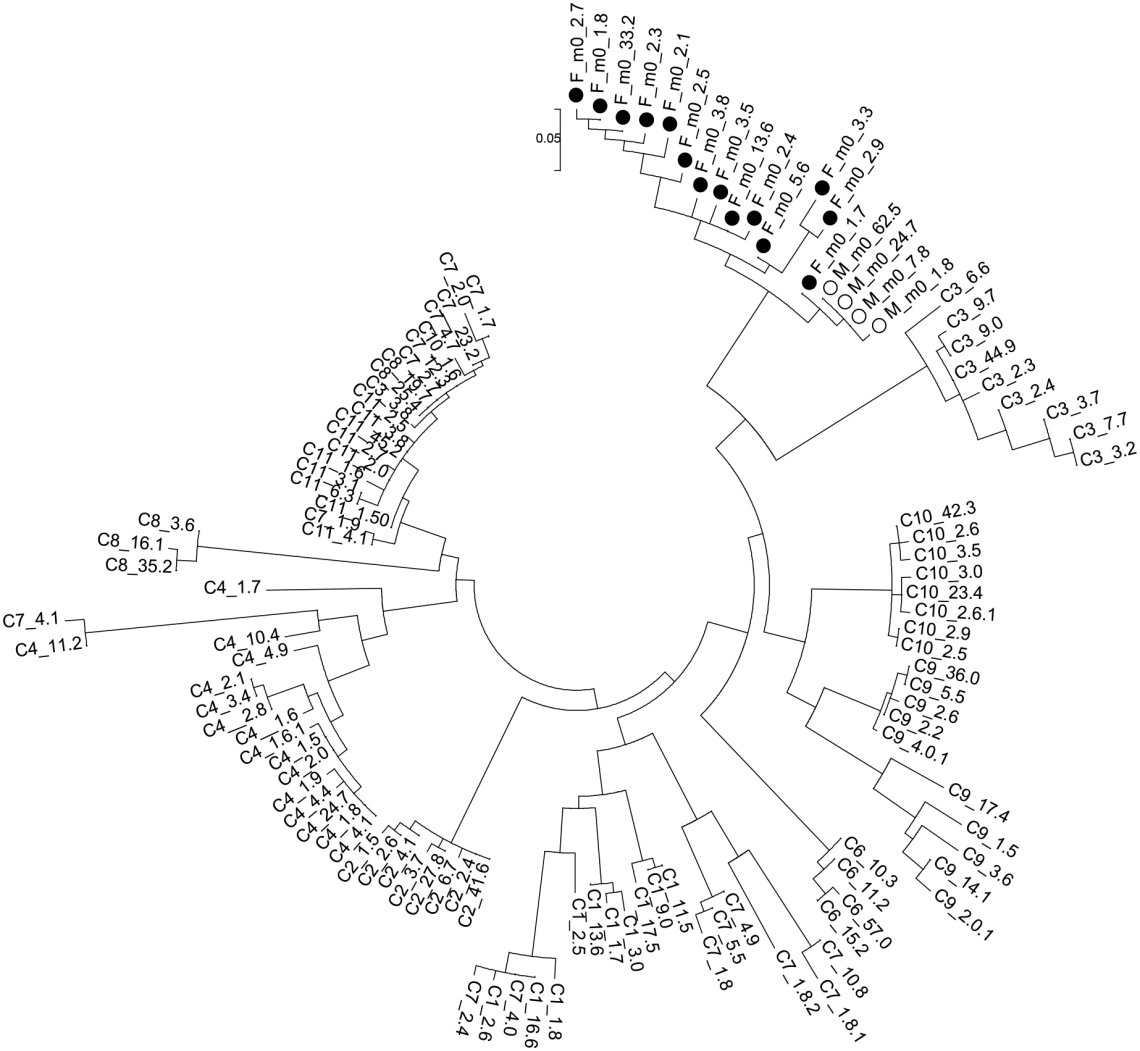
Phylogenetic analysis of HVR1 variants present in both spouses at baseline (m0) and 10 unrelated patients. F, black dot denotes female spouse (F), while the open circle denotes the male spouse (M). Unrelated patients come from the same area and time and were infected with the same HCV subtype 1b. Variant frequencies are expressed as percent values and follow time point of sample collection. The evolutionary history was inferred by using the Maximum Likelihood method based on the Tamura-Nei model [29]. Evolutionary analyses were conducted using MEGA 5.0 [28].

Baseline viral variants from both spouses were compared to sequences from 10 non-related chronic hepatitis C subjects (Fig 3). As seen, donor and recipient variants clustered together and were divergent from HCV variants in all 10 unrelated subjects. Mean distances between these control populations and those of the recipient were 0.280, 0.274, 0.294, 0.292, 0.341, 0.276, 0.316, 0.322, 0.242 and 0.211.

### Evolution of HVR1 epitopes

**Fig 4:**
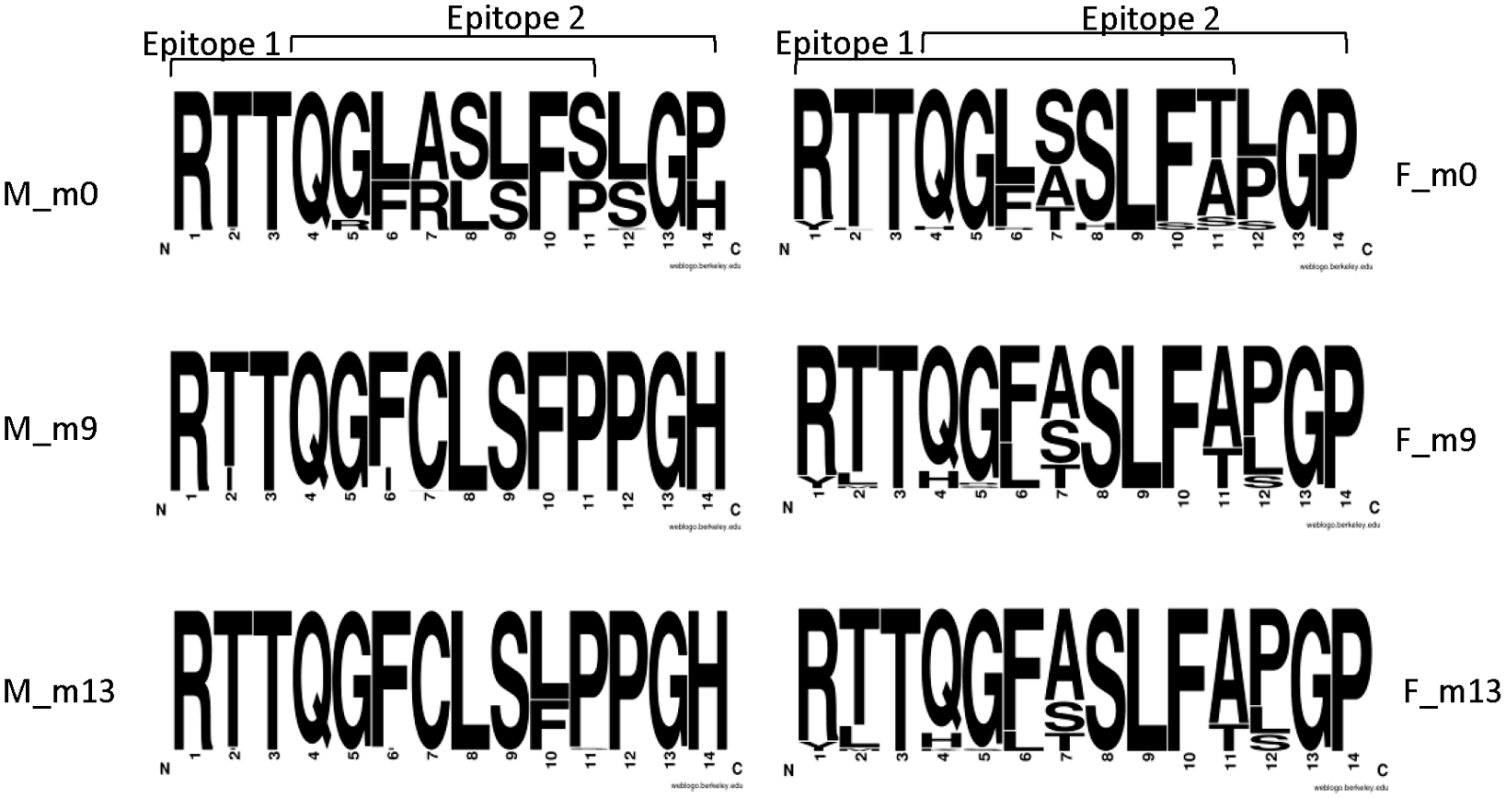
Amino acid sequence logos of HVR1 epitopes generated from populations of HVR1 sequence variants circulating in the male (M) and female (F) spouse. Serum samples were collected at baseline (m0), and at 9 (m9) and 13 months (m13). The male spouse was treated for 6 months with interferon starting immediately after the baseline serum was drawn, but the therapy was ultimately unsuccessful. Epitope 1 comprises positions 1–11 (codon positions 394–404) and epitope 2 comprises positions 4–14 (397–407) [32, 34]. Height of letters within the stack indicates the relative frequency of each amino acid at this position. The analysis was conducted on variants ≥ 1.5% cut-off.

Close to the C-terminus of HVR1 amino acid sequence, two overlapping epitopes for neutralizing antibodies were described, encompassing amino acid positions 394–404 (Epitope 1) and 397–407 (Epitope 2) [32, 34]. In the recipient, the majority of these positions (57.1%) were homogeneous, i.e. composed of only one amino acid across the population. The remaining positions were heterogeneous: positions 2, 5, 6, 7, 8, 9, 11, 12, 14 at month 0, positions 2, 6, 7, 10 and 14 at month 9, and positions 2, 6, 10, 11 at month 13. Over time, nine positions were found to be unstable (change in amino acid composition: positions 5, 6, 7, 8, 9, 10, 11, 12 and 14 (Fig 4).

In the donor, 40.5% of positions were homogenous. Nine positions were heterogeneous at baseline (positions 1, 2, 4, 6, 7, 8, 10, 11, 12), eight at month 9 (positions 1, 2, 4, 5, 6, 7, 11, 12) and eight at month 13 (positions 1, 2, 4, 5, 6, 7, 11 and 12). Over the time of follow up, seven positions were unstable (positions 2, 4, 5, 6, 8, 10, 11).

## Discussion

The increased risk of transmission from HCV-positive patients to household members, including siblings, parents, offspring as well as homo- and heterosexual partners, has been well documented [35–40]. It is of note, that the intrafamilial exposure to infection is high, as over 50% of the seronegative sexual partners of hepatitis C patients develop a specific cellular immune response against the virus without seroconversion or HCV-RNA presence in serum [41].

The actual prevalence of HCV among family members of infected patients was found to be very diverse ranging from 1.3% to 36.4% and depends on the studied population [38, 42]. In the majority of epidemiological studies, no analysis was done to confirm that partners were indeed infected with the same virus [36, 42–46], and even genotyping was done only occasionally [37, 39, 47]. However, genotyping is largely unreliable as it is likely to be similar for a given population in a certain area. Confirmation that a horizontal transmission event has occurred could be demonstrated by a high homology between the respective HCV genomes. Nevertheless, few of the previous studies included phylogenetic analysis [48–51] and sequence comparison was done only on the consensus sequence level [5, 51–53].

In our study phylogenetic analysis has demonstrated much more similarity between HCV strains in spouses than between unrelated subjects, supporting the occurence of intrafamilial transmission [8]. To the best of our knowledge, this study is the first using NGS to demonstrate intrafamilial spouse-to–spouse HCV transmission by a minor frequency variant. Already at baseline (acute infection in the male recipient), no identical HVR1 variants were present in spouses. However, high similarity (98.3%) of one out of the minor donor variants (1.7% frequency) to the most abundant recipient variant, as well as phylogenetic linkage and low interhost distance of baseline HVR1 populations when compared with 10 unrelated patients imply a common ancestry of donor and recipient variants. Indeed, the molecular clock analysis suggested a divergence from a common ancestor 1.5 months prior to baseline, which is compatible with the timing of infection.

Importantly, this minor donor variant would have been overlooked with the use of classical Sanger sequencing approach as it would require sequencing of 175 clones in order to detect a variant of 1.7% frequency with 95% probability. This observation is of special importance for transmission studies, because relationship traits between viral populations may be rapidly lost, especially when using Sanger-based techniques with the lowest detectable variants typically having ≥ 10–20% of frequency [18, 54, 55].

It must be noted that the frequency of the variant putatively transmitted to a new host (1.7%) was close to the applied 1.5% cut-off. This particular cut-off value corresponds to the error rate of the amplification and sequencing procedures determined previously by analysing of cloned HVR1 [27]. Of note, HVR1 and 5’UTR contain several homopolymeric regions (consecutive repeats of identical bases) and pyrosequencing chemistry is highly susceptible to errors at these regions [27, 56]. Importantly, despite repeating the analysis without application of the cut-off, no common HVR1 variants were detectable in both spouses.

The baseline HVR1 population found in the recipient was very narrow which is compatible with the bottleneck phenomenon and is consistent with some other studies in which only a single variant established infection [10, 18, 19]. In our study, the putative infecting variant constituted a small minority of the donor variants (1.7% of frequency) which suggests it may have had some major advantage in the new host. Interestingly, it contained high basic amino acid residues content (29.6%, data not shown) and the presence of basic residues in HVR1 have been previously shown to aid viral entry [57].

Despite the high sensitivity of NGS, it is impossible to determine the exact transmission route, but both sexual and household transmissions seem plausible. We collected cervical-vaginal lavage (CVL) to verify the presence of viral RNA, but neither 5’UTR nor HVR1 could be amplified, which is in line with some previous finding of low prevalence of HCV RNA in genital tract of HCV-monoinfected women [58]. Interestingly, among couples in long-term monogamous heterosexual relationships, the risk of sexual transmission of HCV has been assessed to be very low (0–0.6% per year) [59, 60] and has been even found to be null in a recent metaanalysis of more than 80 studies [61].

Molecular evolution of HCV HVR1 was strikingly different in the chronically infected donor and acutely infected recipient. Recipient's population showed increased complexity (number of variants) over time as well as change in variants composition, with only a few variants dominating the population. This change, called selective sweep, could be the result of selection pressures and is common for such pathogens as influenza [62], HCV [63, 64] and HIV [65]. Furthermore, there was a marked turnover of amino acids within the analyzed epitopes for neutralizing antibodies over time. This observation is compatible with immune selection pressure in acute infection.

After an unsuccessful antiviral treatment was attempted, the composition of variants was entirely changed (baseline *vs* 9 months). While interferon activates cellular rather than humoral response and thus would be expected to have a limited impact on the variability of the HVR1, rapid evolution of this region during therapy is common and predictive with respect to outcome [66–69]. However, a similar change occurred also between month 9 and month 13 when the patient was not receiving any antiviral treatment.

In contrast, the majority of female spouse variants were present in subsequent samples, most of them at a higher frequency. This could be due to virus adaptation and/or limited immune pressure possibly due to immune exhaustion, which is common in chronic HCV infection [70]. Similarly, amino acid composition within two HVR1 epitopes was largely conserved over time.

The 5’UTR sequence remained relatively stable, probably due to lack of selective pressures.

In conclusion, it seems that intrafamilial HCV transmission may be established by a very minor variant and thus the investigation of this phenomenon requires high-sensitivity assays, such as NGS.
